# Evaluation of lipid homeostasis in the late gestational period of rats exposed to dexamethasone

**DOI:** 10.1186/1758-5996-7-S1-A74

**Published:** 2015-11-11

**Authors:** Katia Motta, Marina Fernandes Ruiz, Silvana Bordin, Alex Rafacho

**Affiliations:** 1 Universidade Federal de Santa Catarina, Florianópolis, Brazil

## Background

Glucocorticoids (GCs) may be prescribed under restriction during pregnancy to the treatment of inflammatory-related diseases or to ensure the proper development of the fetus in the preterm delivery. GC treatment is known to cause several metabolic side effects including dyslipidemia. There are some evidences for increased plasma triacylglycerol (TAG) levels during the late period of normal gestation in rats.

## Objectives

We aimed to elucidate whether dexamethasone administration during the late period of gestation alters lipid homeostasis.

## Materials and methods

Adult female Wistar rats received dexamethasone (0.2 mg∙kg-1∙day-1) diluted in the drinking water from days 14 to 19 of gestation (PD) or at equivalent days in non-preganant/virgin rats (nPD). Pregnant (PC) and non-pregnant/virgin (nPC) controls were maintained with water ad lib. Blood glucose and TAG levels were determined before (day 13) and after dexamethasone treatment (day 20). An oral lipid tolerance test (oLTT) was performed at day 20 of gestation in a separate group of rats.

## Results

Partial Results demonstrated that dexamethasone treatment induced significant weight loss in virgin rats and abolished the weight gain in the pregnant rats (p<0.05). Dexamethasone treatment also reduced food intake in both pregnant and non-pregnant rats compared with their control groups (p<0.05). It is also observed a significant decrease in fasting glycemia in pregnant groups compared with non-pregnant rats at days 13 and 20 of gestation (p<0.05 only in PC group). Fasting TAG levels were similar between all groups at day 13 of gestation, but at day 20 of gestation pregnant rats showed a significant hypertriacylglyceridemia compared with non-pregnant rats; an effect that was more pronounced in PD vs. PC group (p<0.05). Are-under-curve obtained through the oLTT revealed lipid intolerance in the pregnant vs. non-pregnant rats, but the AUC were similar between PD and PC groups. Rats treated with dexamethasone exhibited higher TAG values along oLTT compared with their controls (p<0.05).

## Conclusion

We conclude that dexamethasone treatment in the late period of gestation impairs weight gain and exacerbates the hypertriacylglyceridemia and lipid intolerance caused by pregnancy. Additional studies focusing on molecular mechanisms-related to these side effects merit investigation. These data highlights the importance of individual monitoring while dexamethasone is administered in the gestation.

